# The histone methyltransferase *Ezh2* restrains macrophage inflammatory responses

**DOI:** 10.1096/fj.202100044RRR

**Published:** 2021-08-31

**Authors:** Gareth B. Kitchen, Thomas Hopwood, Thanuja Gali Ramamoorthy, Polly Downton, Nicola Begley, Tracy Hussell, David H. Dockrell, Julie E. Gibbs, David W. Ray, Andrew S. I. Loudon

**Affiliations:** ^1^ Faculty of Biology, Medicine and Health University of Manchester, Manchester Academic Health Sciences Centre Manchester UK; ^2^ Manchester University NHS Foundation Trust, Manchester Academic Health Science Centre Manchester UK; ^3^ Department of Infection Medicine and MRC Centre for Inflammation Research University of Edinburgh Edinburgh UK; ^4^ NIHR Oxford Biomedical Research Centre, John Radcliffe Hospital Oxford UK; ^5^ Oxford Centre for Diabetes, Endocrinology and Metabolism University of Oxford Oxford UK

**Keywords:** EZHZ, inflammation, lung, macrophage, neutrophil

## Abstract

Robust inflammatory responses are critical to survival following respiratory infection, with current attention focused on the clinical consequences of the Coronavirus pandemic. Epigenetic factors are increasingly recognized as important determinants of immune responses, and EZH2 is a prominent target due to the availability of highly specific and efficacious antagonists. However, very little is known about the role of EZH2 in the myeloid lineage. Here, we show EZH2 acts in macrophages to limit inflammatory responses to activation, and in neutrophils for chemotaxis. Selective genetic deletion in macrophages results in a remarkable gain in protection from infection with the prevalent lung pathogen, pneumococcus. In contrast, neutrophils lacking EZH2 showed impaired mobility in response to chemotactic signals, and resulted in increased susceptibility to pneumococcus. In summary, EZH2 shows complex, and divergent roles in different myeloid lineages, likely contributing to the earlier conflicting reports. Compounds targeting EZH2 are likely to impair mucosal immunity; however, they may prove useful for conditions driven by pulmonary neutrophil influx, such as adult respiratory distress syndrome.

**Abbreviations**
ARDS: adult respiratory distress syndrome
BMDMs: bone marrow‐derived macrophages
CFU: colony‐forming units
EAE: experimental autoimmune encephalomyelitis
EZH2: enhancer of zeste homolog 2
LPS: lipopolysaccharide
PRC2: polycomb repressive complex 2
TLR: toll‐like receptor

## 1 INTRODUCTION

Macrophages are widely distributed through vertebrate tissues and play essential roles in homeostasis, and in response to injury. Therefore, it is unsurprising that these cells have been implicated in the pathogenesis or host response to a wide‐range of prevalent human diseases, including fibrosis, osteoporosis, obesity, type 2 diabetes, and atherosclerosis.^1^, ^2^, ^3^ Activation of macrophages is critical for an appropriate response against an invading pathogen. For example, alveolar macrophages (AM) located in the alveolar spaces of the airways provide the first line of defense against respiratory pathogens, such as the gram‐ positive bacteria *Streptococcus pneumoniae*.^4^ Recognition of bacteria, and many other pathogens, is facilitated by pattern‐recognition receptors (PRRs) such as toll‐like receptor (TLR). TLRs are a group of cell‐surface and endosomally located PRRs, which, in conjunction with adaptor proteins, activate signaling cascades to induce a cellular response. Multiple TLRs, through MyD88‐dependent, and independent cascades converge to activate NFkB, AP‐1 and IFN regulatory factors (IRFs).

Epigenetics, chromatin modifications which alter DNA availability, has been known for many years to be crucial in the formation of a functional immune response.^5^ Recently, attention has focused on the role of methylation of histone3Lys27 (H3K27), mediated by the polycomb repressive complex, comprising SUZ12, EED and EZH2, with EZH2 as the catalytic subunit. PRC2 is a powerful determinant of differentiated cell state, and by silencing gene expression, limits trans‐differentiation. EZH2 has been intensely studied in cancer,^6^, ^7^ and has gained attention in generating a functional, adaptive immune response.^8^, ^9^ Macrophage switching between functional states has been identified to be dependent on reversible trimethylation of histone H3K27.^10^

Several recent studies have employed genetic targeting of EZH2 or pharmacological EZH2‐selective inhibitors to demonstrate an important role in the regulation of inflammatory responses. These investigations have yielded contradictory results. Pro‐inflammatory outcomes were documented following suppression of EZH2 in inflammatory bowel disease models^11^, ^12^, ^13^ and Kras‐driven lung cancer inflammation.^14^ In contrast, Zhang et al demonstrated that EZH2 in the macrophage drives experimental autoimmune diseases including experimental autoimmune encephalomyelitis and colitis. In this latter study, loss of EZH2 reduced inflammatory responses to stimulus and so reduced disease severity, pointing the way to pharmacological inhibition of EZH2 as a therapeutic strategy in multiple sclerosis.^15^ These discrepancies may result from differential impacts on different cell types, and gene expression programs involved in immune response elaboration, and require further analysis.

Here we show that the actions of EZH2 in the myeloid lineage are complex, inhibiting the proinflammatory responses of macrophages, and enabling efficient neutrophil migratory capacity. This dichotomy of action may also explain the apparent contradictory outcomes of earlier studies of the role of EZH2 in inflammation. The complex actions of EZH2 on innate immune responses are important to consider with the availability of drugs targeting this epigenetic regulator in patients for cancer indications, and suggestions of a role in managing autoimmune disease such as multiple sclerosis.

## 2 MATERIALS AND METHODS

### 2.1 Animal studies

All experimental procedures were carried out in accordance with the Animals (Scientific Procedures) Act of 1986. Male C57BL/6J mice aged 8‐12 weeks were purchased from Harlan Laboratories, UK *Ezh2flox/flox* mice possessing loxP sites upstream and downstream of the 2.7 kb SET domain and were a gift from Alexander Tarakhovsky (Rockefeller, New York). These mice were bred with *LysMcre/+*, a cre recombinase under the control of lysozyme M, to target *Ezh2* for deletion in myeloid cells, including macrophages and neutrophils. Offspring produced were either *Ezh2flox/flox* with no *LysM‐cre* present, these mice acted as littermate controls, or were positive for the cre driver. For all in vivo experiments, conditional targeted cre positive mice were sex and age matched with floxed/floxed littermate controls; females aged 10‐14 weeks were used for in vivo pneumococcal experiments.

In order to genotype these mice and elucidate the correct l*oxP* sites had been inserted the following primers were used for *Ezh2*:

Reverse 1: 3ʹ of loxp: 5ʹ‐AGG GCA TCA GCC TGG CTG TA‐3ʹ
Forward 2: 5ʹ of loxp: 5ʹ‐TTA TTC ATA GAG CCA CCT GG‐3ʹ
Forward 3: left loxp: 5‐ACG AAA CAG CTC CAG ATT CAG GG‐3ʹ

Primers were used at a final concentration of 2.5 μM and were genotyped using the GoTaq Hot Start Polymerase (Promega). The PCR product produced using these primers was run on a 2% agarose gel at 45 volts for 3 hours and mice homozygous for the loxP sites presented a band at 731 bp and those wild type for loxP expression with a band at 691 bp. Mice homozygous for the flox were selected and genotyped for the expression of LysM‐cre using primers diluted to 2.5 μM:

Forward: 5ʹ‐CTTGGGCTGCCAGAATTCTC‐3ʹ
Reverse: 5ʹ‐CCCAGAAATGCCAGATTACG‐3ʹ

The presence of the *LysM‐cre* gene was confirmed by a band at 700 bp.

To target macrophages exclusively and avoid broad myeloid targeting, *Ezh2^fl/fl^* mice were bred with *CX3CR1cre/+* mice. *Ezh2* genotyping was performed as stated. *CX3CR1 cre/+* presence was determined using primers diluted to 2.5 μM:

Forward: 5ʹ‐CCC CAA TGT TCA ACA TTT GC‐3ʹ
Reverse: 5ʹ‐GGA CTG GGG ATG TGG GAG‐3ʹ

cre positive mice were determined by the presence of a band at 500 bp.

For investigations into the role of EZH2 in airway epithelium cells, mice possessing the cre driver *CCSPicre* ^16^ were crossed with mice possessing the *Ezh2flox/flox allele*. The presence of *loxP* sites in *Ezh2* was confirmed as before. *CCSPicre* was evaluated using primers at 2.5 μM:

Forward: 5ʹ‐TCTGATGAAGTCAGGAAGAACC‐3ʹ
Reverse: 5ʹ‐GAGATGTCCTTCACTCTGATT‐3ʹ

and mice were selected as cre positive if a band was identified at 500 bp.

### 2.2 Bone marrow‐derived macrophages (BMDM)

C57BL/6 mice were obtained and sacrificed via cervical dislocation. The hind legs were removed, the muscle and skin dislodged using gauze and to clean the bone before being placed in ice cold sterile Dulbecco's phosphate‐buffered saline (PBS), and this procedure was repeated with the fore legs. The bones were then placed in ice‐cold DMEM (Sigma) with 10% fetal bovine serum (FBS) (Life technologies) and 1% penicillin/streptomycin (Sigma) in a primary tissue culture hood. The ends of the bones were removed and a 17‐gauge needle was used to flush out the bone marrow using the culture media. The bone marrow was disrupted and centrifuged at 1600 rpm for 6 minutes and the supernatant removed. Cells were resuspended in 5‐mL culture medium plus macrophage colony‐stimulating factor (M‐CSF) (Affymetrix eBioscience) at a concentration of 50 ng/mL and counted on a Nucleocounter NC‐250. Cells were then plated into either 6‐well plates at a concentration of 1.5 × 106, 12 well plates at 0.8 × 106, or 24‐well plates at 0.4 × 106/mL and covered with 1.5 mL of culture media + M‐CSF. Cells were kept in a primary incubator at 37℃ and 5% CO_2_ unless otherwise stated and media was changed every 3 days by washing in PBS and replacing culture medium.

### 2.3 PECS

Cells were isolated by washing the peritoneal cavity with ice‐cold PBS several times and collecting the washout. This was then centrifuged at 200 G for 5 minutes at 4℃ and the cells resuspended in DMEM/10% FBS/1%penicillin/streptomycin. Cells were then counted and plated at a concentration of 1 × 10^6^/mL in 6‐well plates for 24 hours. Nonadherent cells were washed away with prewarmed (37℃) DMEM. The remaining adherent cells were termed peritoneal macrophages.

### 2.4 AM

Cells were purified by culling a mouse by cervical dislocation, with care taken to avoid compromising the trachea. A 23‐gauge needle was used to pierce the trachea and fill the lungs with 1 mL ice‐cold BAL (Bronchoalveolar lavage) fluid (PBS, 1% FBS/0.5 mM EDTA). BAL fluid was pulled back from the lungs and placed in an Eppendorf on ice. Eppendorfs were centrifuged at 200 G for 5 minutes at 4℃ and the pellet was resuspended in DMEM (10% FBS/1% penicillin‐streptomycin). Cells were plated out in 24‐well plates and cells were left to adhere for 24 hours before the removal of non‐adherent cells by washing in pre‐warmed (37℃) DMEM. The remaining adherent cells were termed AMs.

### 2.5 Neutrophil isolation

Hind limbs were isolated as above. The bone marrow was flushed through and was counted and resuspended at 1 × 107/mL with 5% normal rat serum (EasySep) in PBS and was incubated on ice for 15 minutes. Mouse neutrophil enrichment cocktail (EasySep) containing primary antibodies against non‐neutrophils was added at 100 μL/mL and incubated on ice for 15 minutes. Bone marrow was washed in 10 mL of PBS, centrifuged at 8000 rpm for 5 minutes and resuspended at 1 × 10^7^/mL in pbs. Magnetic secondary antibodies were added at 100 μL/mL, vortexed, and placed on ice for 15 minutes. The sample was placed in a magnetic field for 2 minutes at room temperature and the remaining PBS containing the neutrophils was removed.

### 2.6 Adoptive transfer

Neutrophils were isolated, as described above, from PEP3 mice that possess a naturally occurring CD45.1 isoform as opposed to CD45.2 found in C57BL/6 mice. Neutrophils were diluted (5 × 10^6^ neutrophils/mL) in saline and 200 μL was injected into the tail. Mice were left for 1 hour to allow neutrophil circulation prior to any experiments taking place.

### 2.7 Intraperitoneal LPS

LPS (Sigma) was reconstituted in saline and mice were injected using a 23‐gauge needle at a dose of 1 mg/kg in 200 μL. After injections, mice were returned to their home cages to generate an immune response in the peritoneal cavity for a time period stated in each individual experiment. Macrophage activity was evaluated by isolating the cells from the peritoneal cavity as described.

### 2.8 Aerosolized LPS

Mice were placed in individual restraining compartments of an inExpose chamber (Scireq). The lid of the compartment was then securely placed on the chamber, placed in a fume hood and a nebulization unit (Scireq) was attached to the chamber. Saline or LPS was reconstituted in saline (2 mg/mL) and was then placed in the nebulization unit. The flow rate of the machine was set at 4 L/minute and the nebulizer was turned on to aerosolize the LPS. Mice were then exposed to the aerosolized LPS or saline control for twenty minutes. Mice were then returned to their cages for 5 or 24 hours before being culled by intraperitoneal overdose of pentobarbitone or by cervical dislocation.

### 2.9 BAL

After mice were euthanized, the trachea was exposed by blunt dissection and a 23‐gauge needle, connected to a 1‐mL syringe filled with BAL fluid, was inserted into the trachea. The BAL fluid was then forced into the lungs by pressure and was left for 1 minute. BAL fluid was then extracted from the lungs and then fluid was placed in an Eppendorf on ice.

### 2.10 Cell counting

Cells were pelleted after in vivo experiments and were resuspended in 1 mL PBS. Acridine Orange provides a total cell count while DAPI provided information on the total number of dead cells allowing the calculation of viability of each sample using NucleoView software for Nucleocounter.

### 2.11 Exposure to ***S pneumoniae***

*S pneumoniae* strain D39 (National collection of Type Cultures 7466) was prepared in Todd Hewitt Broth (Sigma) and incubated at 37℃ 5% CO_2_ until an optical density of OD600 was achieved from 10 mL of growth media. Mice were then placed under light anesthesia using isoflurane. While under light anesthesia, the mice were scruffed and, using a pipette, 100 μL of *S pneumoniae* was dropped onto the nose to intranasally administer 2 × 10^4^ colony‐forming units of D39. Mice were closely monitored and weighed every 12 hours to assess health (a threshold of 20% weight loss was used as an end‐point) and were culled 48 hours after exposure by intra peritoneal pentobarbitone overdose in a category 2 fume hood. To evaluate the infective burden, 100 μL of peripheral blood was obtained immediately after cull and placed in 5‐μL heparin (Sigma) to prevent clotting and was placed on ice. The chest cavity was then opened and the lungs were exposed. All lobes of the lung were removed and placed in ice‐cold DMEM and placed on ice.

### 2.12 Culture of *S pneumoniae*

To understand the infective burden in each animal, blood and lung tissue were obtained as above. Lung tissue was digested as described in Section 2.15. Serial dilutions of blood and lung tissue were then made in 96‐well plates. A volume of 20 μL of the serial dilution of blood or lung digest was then placed onto horse‐blood agar plates in duplicate for each sample. Blood agar plates were produced by making a 3.9% Columbia blood agar base (Sigma) in deionized water and autoclaving. Agar was allowed to equilibrate to 50℃ in a water bath before 5% defibrillated horse blood (OXOID) (TCS Biosciences) was added. The solution was then poured into 10‐cm plates and allowed to set at 4℃. Once bacteria was added to the agar plates, they were left to air‐dry in a cell culture hood for 30 minutes before being placed in a 37℃ incubator at 5%CO_2_ for 24 hours. Blood agar plates were then removed from the incubators and the bacterial growth was counted at each serial dilution to understand the bacterial growth in the blood and lung of each mouse.

### 2.13 Lung digestion

The left lobe of the lung was placed into a bijou containing ice‐cold DMEM during the cull. DMEM was removed and the lung lobe was then chopped into small sections using dissection scissors. 1 mL of digestion media: DMEM containing 2.7% Liberase (Roche) and 0.05% DNase I (Roche) was added to each bijou and they were placed in a shaking incubator at 37℃ for 30 minutes. To stop the digestion, 0.005 mM EDTA in DMEM was added to the digestion media in an equal volume. The digestion was then forced through a 70‐µm culture sieve. At this point, samples required for *S pneumoniae* infection analysis were taken and placed on blood agar plates.

### 2.14 RNA extraction from tissue

RNA was isolated from tissues using Trizol reagent (Sigma).

### 2.15 RNA analysis

RNA was isolated from cells using the RNeasy mini kit (Qiagen).

### 2.16 Reverse transcription

All samples of RNA were treated with 1 μL of RQ1 DNase (Promega). DNase treated RNA was converted to cDNA using the RNA‐cDNA kit (Applied Biosystems) in accordance with the manufacturer's instructions.

### 2.17 Quantitative real‐time PCR (qPCR)

cDNA was analyzed in 96‐well MicroAmp optical reaction plates (Life Technologies). All reactions were performed in a StepOnePlus system (Life Technologies). Relative expression of genes was determined using the threshold cycle comparative CT. Gapdh was used as a housekeeping gene and gene expression was normalized to the housekeeping gene and relative quantification values were calculated in relation to a reference sample, which is denoted in each experiment. Fold change was determined using the formula: 2–Δ ΔCT = 2–(ΔCt (experimental sample) – ΔCt(control)). Samples were run in either triplicate or duplicate.

### 2.18 Histone purification

Histones were purified from cells by growing macrophages to a density of 3 × 10^7^ (Active Motif ‐ 40025). Histone H3K27me3 conversion was measured using the Active Motif kit (Epiquik Histone Methyltransferase activity assay kit (P‐3005)).

### 2.19 MTT viability assay

Cell viability after treatment with EZH2 inhibitors was determined using an MTT assay.

### 2.20 Enzyme‐linked immunosorbent assay (ELISA)

The concentration of multiple proteins was determined using ELISA in a variety of samples including BAL, peripheral serum, and lung homogenate. All ELISA kits were DuoSet and were obtained from R&D systems, and run according to the manufacturer's instructions.

### 2.21 Nanostring

RNA was then run on a Nanostring nCounterTM (Nanostring Technologies) in conjunction with the premade CodeSet called mouse inflammation panel V2 (Nanostring Technologies).

### 2.22 Transcription factor activation assays (TransAM)

RelA and IRF‐3 transcription factor activation assays (Active Motif) were carried out using the same protocol with the only difference being the 96‐well plates and the antibodies used, each of which corresponding to the respective kit, Rela: 40096, IRF‐3:48396.

### 2.23 Pharmaceuticals

GSK343 (Selleckchem) was resuspended in DMSO and cells were treated at a concentration of 200 nm. GSK126 (Sigma) was resuspended in DMSO and cells were treated at 200 nm. UNC1999 was used in both in vitro and in vivo studies. For in vitro dosing, UNC1999 was reconstituted in DMSO and was used on cells at 2 µM concentrations. For in vivo work, UNC1999 was reconstituted in vehicle (0.5% of sodium carboxymethylcellulose and 0.1% of Tween 80 in sterile water) using constant agitation for 60 minutes to dissolve the powdered drug. Reconstituted UNC1999 or vehicle was administered to mice by oral gavage at a dose of 50 mg/kg in 100 μL twice daily for 48 hours prior to immunological challenge.

### 2.24 Flow cytometry

1 × 10^6^ cells were placed in a 96‐well plate and cells were suspended in 50 μL PBS containing Zombie UV live: dead stain at 1:2000 (Biolegned) for 15 minutes in the dark at RT. Live: dead staining was halted by adding 150 μL FACS buffer (PBS/4% FBS) and centrifuging at 2000 rpm for 2 minutes at 4℃. Supernatant was flicked off and cells were washed in 200 μL FACs buffer and centrifuged as stated. The pellet was resuspended in 50 μL fc‐block (CD16/CD32 antibody at 1:50 in FACS buffer) and incubated at RT for 10 minutes; 150 μL of FACS buffer was then added to each well and the plate was centrifuged as before. The pellet was then resuspended 50 μL primary antibody cocktail (Table 1) or FMO cocktails for 25 minutes in the dark; 150 μL FACS buffer was added to each well and plates were centrifuged, and then washed three times. If a secondary antibody was required, it was added to FACS buffer 1:5000 and the pellet was resuspended in 50 μL of FACS buffer with secondary antibody for 25 minutes at RT in the dark. Plates were then washed three times before the addition of 50 μL 1% formaldehyde in FACS buffer for 5 minutes at RT in the dark. Plates were then washed twice in FACs buffer and the pellet was resuspended in 200 μL FACs buffer. The samples were then acquired on either a BD Fortessa or BD LSR‐II using BD‐FACSDivaTM software (BD Biosciences).

**TABLE 1:** Flow cytometry antibody‐fluorophore and dilution used for primary antibody cocktail

Fluorophore	Antibody target	Dilution factor
FITC	Ly6G	200
AF700	Ly6C	200
APC/eFluor780	CD3	100
APC/eFluor780	CD19	200
APC/eFluor780	NK1.1	200
APC/eFluor780	Ter119	200
BV421	CD45.2	200
BV650	CD45.1/CD45	200
BV711	CD11b	600
Live/Dead Blue	L/D Blue	2000
PE/CF594	Siglec F	400
PE/Cy7	CD64	100

Fluorophore Antibody target Dilution factor FITC Ly6G 200 A647 MerTK‐Bio 100 AF700 Ly6C 200 APC/eFluor780 CD3 100 APC/eFluor780 CD19 200 APC/eFluor780 NK1.1 200 APC/eFluor780 Ter119 200 BV421 CD45.2 200 BV605 CD45.1/CD45 200 BV711 CD11b 600 200 Live/Dead Blue L/D Blue 2000 PE/CF594 Siglec F 400 PE/Cy5 MHC‐II 10,000 PE/Cy7 CD64 100.

In order to set up the flow cytometers correctly compensation beads appropriate for the panel being analyzed were generated. Compensation beads (BD biosciences) were incubated with each individual primary antibody at the same concentration used on the cells for 10 minutes at RT in the dark. Beads were pelleted by centrifugation at 100 G for 5 minutes. The pellet was resuspended in 200 μL of FACS buffer before acquisition. For Live: dead compensation, ArC reactive beads (Thermo Fischer) were used according to the manufacturer's instructions with 1:2000 Zombie: UV Live: dead dye. Immune cells were identified using FLOWJOTM software and all gates were drawn based on published papers using fluorescence minus one (FMO) controls. First, all single cells were gated on, and debris removed, then live cells were selected, and a lineage gate used to exclude cells with the following markers CD3, CD19, NK1.1 and Ter119. AMs were identified as Ly6G‐, CD64+ Siglec F+, and interstitial macrophage were identified as Ly6G‐, CD64+ Siglec F‐ and Neutrophils were identified as Ly6G+ CD11b+.

### 2.25 Boyden chamber assay

A Neuro Probe AP48 48‐well Boyden chamber (Neuro Probe) was used for all assays. The chamber was prepared by adding 26 μL pre‐warmed (37℃) CXCL‐5 (0, 0.5, 50, 500 ng/mL) or CCL2 (0, 0.5, 50, 500 ng/mL) (R&D systems) in DMEM to the lower chamber. A filter membrane of either 3 μM (neutrophils) or 5 μM (Macrophages) pore size (Neuroprobe) was then placed on top of the lower chamber. The silicone gasket and the upper plate were then placed on top of the membrane and the membranes were sealed together with a thumb nuts. 50 000 cells of interest in 50 μL of DMEM were added to the top chamber avoiding air bubbles. The Boyden chamber was then placed in an incubator at 37℃ and 5% CO_2_ for 30 minute or 2 hours. The media was then removed from the top and bottom chambers and the number of cells was counted to obtain a percentage of cells which migrated into the bottom chamber.

### 2.26 Statistics

Statistical analysis was performed, and graphs were generated using Graphpad Prism (Software, Version 9.0). All data points are shown in their entirety, with the mean presented for parametric data and median for nonparametric. For comparisons between two groups, unpaired t‐test was used for parametric data and Mann‐Whitney U test for nonparametric data. For comparison of continuous variables between more than two groups, ANOVA was used with Tukey's correction for multiple testing for parametric data and Kruskal‐Wallis test was used for nonparametric data with Dunn's multicomparison test. A *P*‐value < .05 was taken as statistically significant.

## 3 RESULTS

### 3.1 Deletion of *Ezh2* increases inflammatory cytokine production in multiple macrophage lineages

**FIGURE 1:**
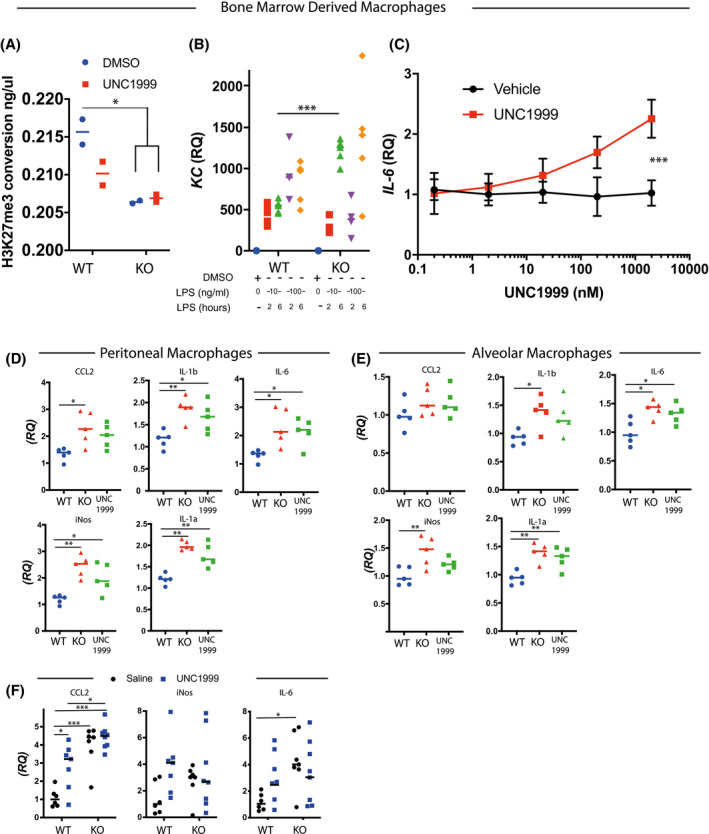
*Ezh2* increases macrophage inflammatory cytokine production. A, Functional assay of EZH2 in nuclear lysates from BMDMs derived from LysM‐Ezh2^fl/fl^ mice treated for 48 hours with either DMSO or 2 μM UNC1999. (n = 4, 2‐way ANOVA, post hoc Tukey's multicomparison test *P < .05, median plotted). B, Isolated macrophages from bone marrow and treated with different doses of LPS for 2 or 6 hours. Ex vivo BMDMs from Ezh2^–/–^ macrophages and littermate controls were treated with LPS for 2 or 6 hours at a dose of 10 or 100 ng/mL and the cytokine response assessed by RNA quantification. (n = 5, median plotted, 2‐way ANOVA, post hoc Tukey's multicomparison test. *P < .05). C, UNC1999 dose response curve for IL6 expression in BMDMs. Ex vivo BMDM cells were incubated with either UNC1999 or vehicle at the indicated concentrations for 48 hours before stimulation with LPS (100 ng/mL) for 2 hours. quantification of IL6 mRNA by qRT‐PCR. (n = 3, 2‐way ANOVA, post hoc Tukey's multicomparison test. ***P < .001). D, Peritoneal macrophages & (E) Alveolar macrophages harvested from EZH2^fl/fl^ (WT) or EZH2^fl/fl^‐LysM (KO) mice and vehicle treated for 1 hour, before to 10 ng/mL LPS for 6 hours. Additionally, cells from Ezh2^fl/fl^ mice were pretreated with 2 μM UNC1999 for 1h prior to LPS (UNC1999). Cytokines measured by qRT‐PCR. (n = 5, median plotted, 2‐way ANOVA, post hoc Tukey's multicomparison test. *P < .05, **P < .01, ***P < .001). F, In vivo responses were assessed in Ezh2^fl/fl^ (WT) or LysM‐Ezh2^fl/fl^ (KO) mice which were pretreated with either vehicle, or 2 μM UNC1999 by an intraperitoneal injection (IP) of 50 mg/kg per day for 2 days before a single IP injection of LPS 1 mg/kg. Peritoneal cells were washed out after 2 hours, and inflammatory mediators were quantified by qRT‐PCR. mRNA was quantified relative to GAPDH, and normalized to vehicle‐treated control mice given LPS. (n = 6‐8, *P < .05 vs vehicle‐treated mice, 2‐way ANOVA, post hoc Tukey's multicomparison test)

We assessed the efficacy of genetic targeting, using cultured BMDMs from *LysM‐Ezh2^fl/fl^* (myeloid‐targeted) mice and showed loss of H3K27 trimethylation (Figure 1A). We then showed loss of *Ezh2* gene expression in alveolar, peritoneal, and BMDMs (Figure S1A). We also employed an EZH2 inhibitor in wild‐type BMDMs (UNC1999), and showed resulting loss of H3K27me3, similar to the genetically targeted cells (Figure 1A).

Next, we generated mice lacking EZH2 in the macrophage lineage, for in vivo infection studies, by using the *CX3CR1*‐cre driver line. We tested the in vitro response of EZH2‐deficient BMDMs to 10 or 100 ng/mL LPS for 2 or 6 hours (Figures 1B and S1A). The pattern of CXCL1 (KC) gene expression revealed a complex interaction between genotype, LPS concentration, and duration of exposure. It appears that the kinetics of response are altered in the absence of EZH2, with a delay in the initial response followed by a delayed, and greater response (Figure 1B). In particular, the responses of CXCL1 (KC) were striking, with duration of LPS exposure exquisitely impacting the LPS induction in the absence of EZH2. This finding is similar to a previous report, but the impact of EZH2 is more complex than a simple gain or loss of global inflammatory response.^15^ We then moved on to test the EZH2 inhibitor UNC1999^17^ on wild‐type BMDMs, which showed a dose‐dependent increase in the expression of *IL‐6* in BMDMs post LPS‐treatment (Figure 1C). We extended these analyses to two further EZH2 inhibitors and found in both cases a dose‐dependent induction of LPS‐induced *IL6* (Figure S2).

Macrophages are found in multiple tissue compartments and exhibit a variety of different phenotypes.^18^ To check the extent of the wider actions of EZH2, we isolated peritoneal macrophages (Figure 1D) and AMs (Figure 1E) from *Ezh2^fl/fl^* (WT) and *LysM‐Ezh2^fl/fl^* (KO) mice. The isolated cells were treated with 10 ng/mL LPS for 6 hours, before analysis of a panel of LPS‐induced inflammatory genes. Here we observed that either genetic loss of EZH2, or pharmacological inhibition with UNC1999, amplified the cytokines responses to macrophage activation. In general, our data show that EZH2 puts a brake on inflammatory responses once it is established, consistently at 6 hours after treatment.

Because the actions of EZH2 on the responses of isolated macrophages from different sites showed some variation, and some of the responses we saw conflicted with earlier publications,^15^ we opted to move to in vivo analyses. Here we treated *LysM*‐*Ezh2* ^fl/fl^ and *Ezh2* ^fl/fl^ mice with either UNC1999 or vehicle for two days before intraperitoneal LPS (1 mg/kg) to activate resident peritoneal macrophages (Figure 1F). Genetic deletion of *Ezh2* in myeloid cells resulted in a target gene selective gain in response to LPS injection, *ccl2*, and *il6* showed significantly increased responses. UNC1999 significantly increased *ccl2* induction, by a small amount in the *Ezh2^fl/fl^* control mice, with no‐significant impact on responses of *iNOS* and *IL6*.

### 3.2 *Ezh2* deletion enhances RelA activation in response to TLR‐4 activation

**FIGURE 2:**
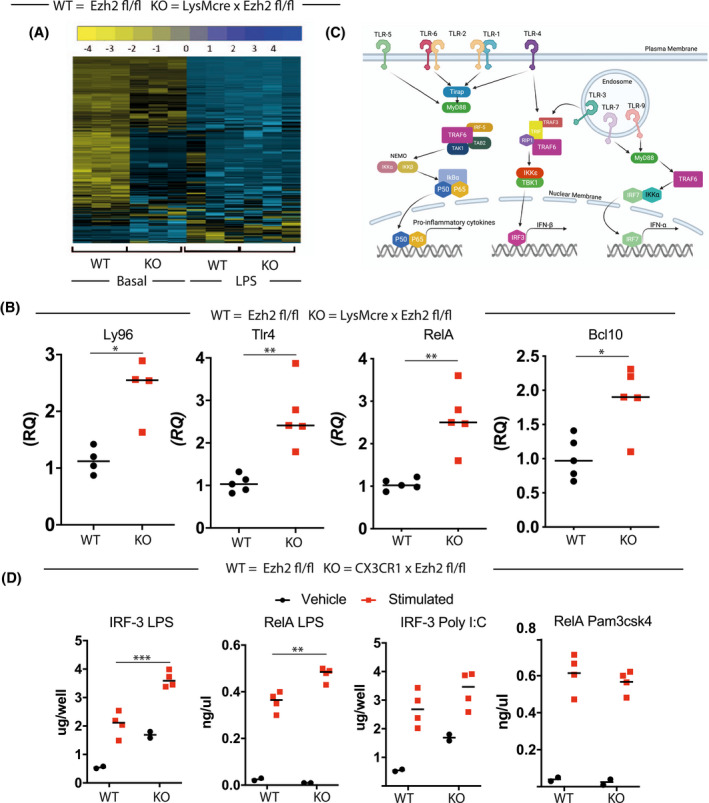
Selective impact of EZH2 deletion on TLR4 signaling. A, To investigate the consequences of EZH2 deletion on immune pathways, a Nanostring analysis was performed comparing BMDMs from LysM‐Ezh2^fl/fl^ (KO) mice with EZH2^f/f^ (WT) littermate controls. This analysis was undertaken using the mouse inflammation panel V2 Nanostring code‐set for inflammatory gene expression. Cells were differentiated in vitro, and analyzed under unstimulated basal conditions. The RCC files (NanoString compressed data file type) were then analyzed in the nSolver^TM^ software package (NanoString Technologies). The heatmap shows the three replicates. B, This transcript levels of Ly96, TLR4, RelA, and BCL10 are plotted. (n = 4‐5, median plotted, Mann‐Whitney *U*‐test *P < .05, **P < .01). C, Diagram of the main TLR signaling pathways, highlighting overlap between TLR1/2, TLR3, and TLR4 cascades. D, To investigate the potential impacts on other TLR pathways, we used a TLR agonist panel to challenge each TLR individually in the absence of EZH2. Cells were incubated with the TLR1/2 ligand Pam3csk4, the TLR3 ligand PolyI:C, or the TLR4 ligand LPS for 2 hours before harvest and analysis using the TransAm system. (n = 2‐4, median plotted, 2‐way ANOVA, post hoc Tukey's multicomparison test. **P < .01, ***P < .001)

To investigate the wider impact of *Ezh2* deletion on expression of immune‐related genes in BMDMs, we used a pre‐made CodeSet (Mouse inflammation panel V2 Nanostring^TM^) (Figure 2A, Table S1). We saw that the major changes in gene expression were between the *Ezh2^fl/fl^*, and the *LysM‐Ezh2* under basal conditions, with LPS exposure of the cells exerting a dominant effect on the gene expression profile (Figure 2A). As many of the differentially regulated genes under basal conditions were components of the TLR‐NFkB system, we targeted some examples (Figure 2B). The TLR signaling system employs a number of overlapping elements (Figure 2C), and so to refine the parts of the cascade functionally affected by EZH2, we used a TransAm assay, which uses DNA‐binding and antibody detection to estimate transcription factor function. Here we were able to show that ligands for TLR4 (LPS) caused increased activation of both NFkB, and IRF3, but ligands specific for TLR3 (PolyI:C), or TLR2 (Pam3csk4) were not impacted by loss of EZH2 (Figure 2D), thereby anchoring the gain of function to TLR4 action.

### 3.3 Targeted deletion of EZH2 in pulmonary macrophages provides protection against bacterial infection

Analyses to date had revealed a complex network of EZH2 effects in myeloid cells, and so to determine the physiological significance, we moved to in vivo testing. To evaluate the in vivo macrophage‐specific responses, we focused on use of the macrophage‐specific Cx3CR1‐cre driver to target EZH2 deletion to macrophage lineages.

The myelomonocytic driver *LysM‐cre* is known to target both macrophage and neutrophil populations, and loss of EZH2 in neutrophils impairs the migratory behavior of these important migratory phagocytes.^19^ However, the *CX3CR1* driver does not affect neutrophils, permitting analysis of the role of EZH2 in macrophages in lung inflammation models, such as aerosolized LPS, in which a neutrophil response is prominent and essential.

**FIGURE 3:**
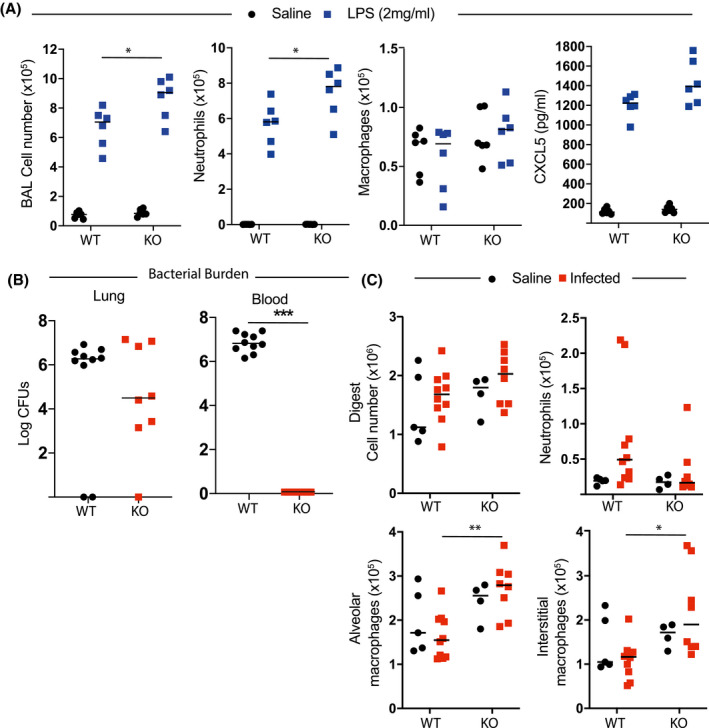
Inflammatory and immune function of EZH2 in macrophages. A, Pulmonary in vivo inflammatory responses in CX3CR1‐Ezh2^fl/fl^ mice. Mice were subject to nebulized LPS, and after 4 hours killed. The bronchoalveolar lavage was analyzed by flow cytometry, and the immune cell fractions are plotted, macrophages (Ly6G‐ CD64+) and neutrophils (Ly6G+ CD11b+). (n = 6, median plotted, 2‐way ANOVA, post hoc Tukey's multicomparison test. *P < .05). B, Pneumococcal responses in Ezh2^fl/fl^ or CX3CR1‐Ezh2^fl/fl^ female mice were compared. All animals were inoculated with pneumococcus, and then analyzed 48 hours later. Bacterial counts (colony‐forming units, CFUs) in the lung and peripheral blood were measured and are plotted. (n = 8‐10, median plotted, Mann‐Whitney *U*‐test *P < .05, **P < .01, ***P < .001). C, Immune cells were recovered from lung digest in animals from (b) and myeloid cell content measured by flow cytometry, alveolar macrophages (Ly6G‐ CD64+ Siglec F+), interstitial macrophages (Ly6G‐ CD64+ Siglec F‐) and neutrophils (Ly6G+ CD11b+). (n = 4‐10, median plotted, 2‐way ANOVA, post hoc Tukey's multicomparison test. *P < .05, **P < .01)

There was a small but significant increase in the total number of cells in targeted mice in BAL derived from the lungs of mice exposed to an aerosol of LPS, which flow cytometry revealed was caused by a significant increase in the number of neutrophils^20^ (Figure 3A). There were no differences in numbers of macrophages or cytokines present within the BAL CXCL5 (Figure 3A), CCL2, IL‐6, or IL‐1a (Figure S3A).

We next assessed responses to bacterial infection, using an intranasal delivered *S pneumoniae* infection model. This is a prevalent bacterial pathogen, which expresses multiple lipoproteins capable of activating TLR2, and also pneumolysin, which may activate TLR4, as well as driving tissue damage and so broadly activating innate immune responses. EZH2‐targeted mice were assessed 40 hours after infection; they were killed at this time point due to the severity of disease. The numbers of colony‐forming units (CFU) recovered from lung did not differ by a statistically significant degree, but there was a trend to reduced colony‐forming units in the EZH2‐deleted animals. This difference by genotype was amplified in the blood compartment with no CFUs detected in blood of targeted mice (Figure 3B). Flow cytometry analysis of the lung digest revealed no difference in the total number of cells/mg of tissue 40 hours after infection. However, the number of interstitial macrophages in the lung after infection was significantly greater in the *Ezh2^fl/fl^ Cx3CR1‐cre* mice (Figures 3C and S3B). Thus, loss of EZH2 in pulmonary macrophages greatly enhances their ability to restrict the systemic spread of a localized infection. This was a really striking phenotype proving a role for macrophage EZH2 in regulating pulmonary bacterial defense, with a gain of function seen in macrophage‐specific *Ezh2* knockout animals. We also tested inhibition of EZH2 in vitro using the GSK343 compound, and measured the impact on macrophage phagocytosis, using our optimized method.^20^ However, there was no effect on macrophage phagocytosis of *Staphylococcus aureus*, and so we did not pursue this further.

### 3.4 *Ezh2* deletion in pulmonary epithelial cells does not affect influenza responses

We have previously shown that pulmonary bronchial epithelial cells play a sentinel role in gating TLR‐4 mediated time‐of‐day responses to LPS and inflammatory responses, regulated by specific elements of the epithelial cell clockwork.^16^, ^21^ Since our data revealed a striking in vivo phenotype following targeting of macrophages, we tested the potential generality of this by targeting EZH2 in bronchial epithelial cells using a *CCSP‐cre* driver.^16^

As the bronchial epithelium is the primary target of the influenza virus, we tested the responses to influenza inoculation. In both targeted and wild‐type animals, there was a marked reduction in body weight and establishment of an inflammatory response (Figure S4A–E), but no effect of genotype on these parameters, histological score, and neutrophil or macrophage cell number. Thus, despite evidence for the importance of the bronchial epithelial cell as a local‐acting stem cell population^16^ and the role of these cells in gating time‐of‐day responses to LPS, targeting of EZH2 has no significant impact on the kinetics of inflammatory responses, nor response to epithelial‐targeted viral infection.

### 3.5 Neutrophil EZH2 is essential to confer protection against bacterial infection

**FIGURE 4:**
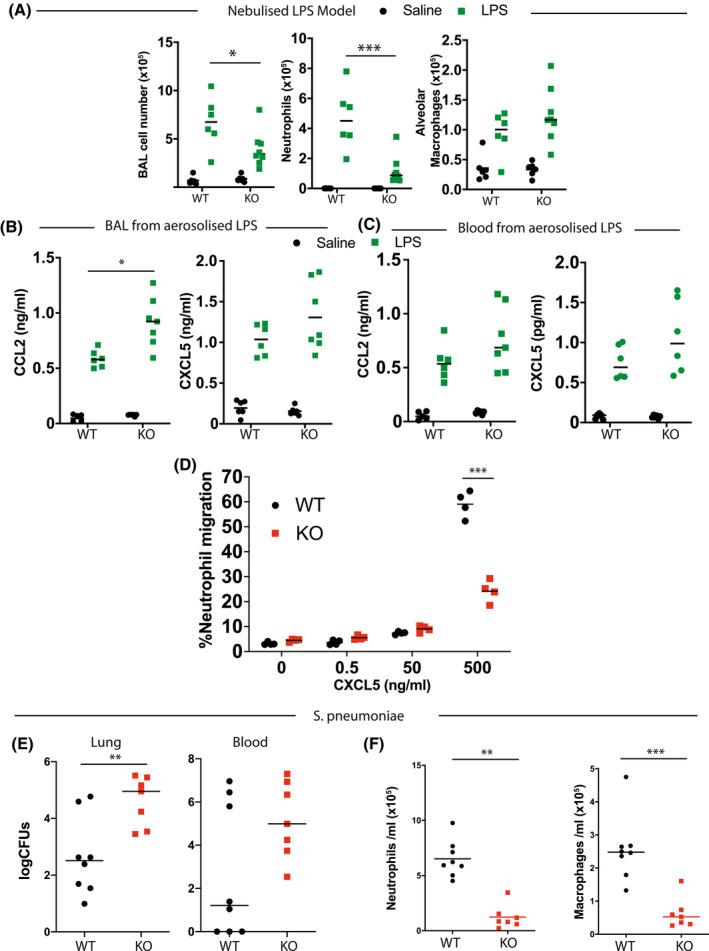
EZH2 differentially impacts myeloid cell types. A, Nebulized LPS was administered to Ezh2^fl/fl^ (WT) or LysM‐Ezh2^fl/fl^ (KO) animals. After 4 hours, Bronchoalveolar lavage (BAL) immune cell content, macrophages (Ly6G‐, CD64+), and neutrophils (Ly6G+, CD11b+) by flow cytometry analysis. (n = 6‐8, median plotted, 2‐way ANOVA, post hoc Tukey's multicomparison test. *P < .05, ***P < .001). B and C, Chemokine content from (A) was determined by ELISA. This revealed an intact chemokine response in BAL (B) or blood (C) from LysM‐ EZH2^fl/fl^ mice (n = 6‐8, median plotted, 2‐way ANOVA, post hoc Tukey's multicomparison test. *P < .05). D, To evaluate neutrophils’ behavior, cells were isolated from the bone marrow of genetically targeted mice and were placed in Boyden chamber across a membrane from a CXCL5 gradient. (n = 4, median plotted, 2‐way ANOVA, post hoc Tukey's multicomparison test. ***P < .001). E, Female mice of the indicated genotype were inoculated with pneumococcus and 48 hours later, the bacterial contents of lung tissue and peripheral blood were measured. Significantly higher CFUs were observed in the lung (n = 7‐8, median plotted, Mann‐Whitney *U*‐test **P < .01). F, Lung immune cell content was measured from BAL by flow cytometry analysis macrophages (Ly6G‐, CD64+) and neutrophils (Ly6G+, CD11b+), postpneumococcal infection. (n = 7‐8, median plotted, Mann‐Whitney *U*‐test **P < .01, ***P < .001)

Previous work has shown that EZH2 is critical for neutrophil extravasation.^19^ To investigate this phenotype further in our physiological models, we tested inflammatory responses of *LysM‐Ezh2^fl/fl^* mice. In this model, all types of macrophage and neutrophil cells are targeted. Initially, we tested pulmonary inflammatory challenges to nebulized LPS exposure. BAL analysis at +4 hours revealed a significant reduction in recruitment of neutrophils, but no significant differences in AM number (Figure 4A), as anticipated. We next assessed whether production of pulmonary neutrophil chemoattractants was disrupted, and analyzed BAL and peripheral blood for LPS‐induced expression of the neutrophil chemoattractant CXCL5 and the monocyte chemoattractant CCL2. This revealed an increase in CCL2 in BAL from *LysM‐Ezh2^fl/fl^* mice following aerosolized LPS challenge, but no difference in blood or CXCL5 from either tissue. (Figure 4B,C). Therefore, it appeared that the defect in neutrophil recruitment may lie with the neutrophil migratory response rather than with a lack of appropriate chemokine signal.

To test this, we used a modified Boyden chamber to measure neutrophil migration against a CXCL5 gradient. This revealed a marked defect in neutrophil migratory response, supporting a major impact of EZH2 on neutrophil motility, likely resulting from the cytoskeletal remodeling effects of EZH2 loss in these cells (Figure 4D), although we did not go on to confirm such changes in our model.

To evaluate the biological relevance of this phenotype in the context of bacterial infection, we exposed *LysM‐Ezh2^fl/fl^* mice to infection with *S pneumoniae*. After 48 hours of infection, we observed a significant increase in the bacterial colonization of the lung, but no change in the recovery of bacteria from lung and peripheral blood (Figure 4E). Lung bronchoalveolarlavage (BAL) revealed a significant reduction in total cell counts, in both neutrophil and macrophage populations (Figure 4F). The reduction in neutrophil recruitment was expected, but the attendant loss of macrophages was a surprise, and likely results from the extent of infection, and inflammation, perhaps associated with macrophage apoptosis as a host defense mechanism, or may result from impaired recruitment and differentiation of circulating monocytes.^22^

### 3.6 Defective neutrophil migration can be rescued by the transplant of *Ezh2* intact neutrophils

**FIGURE 5:**
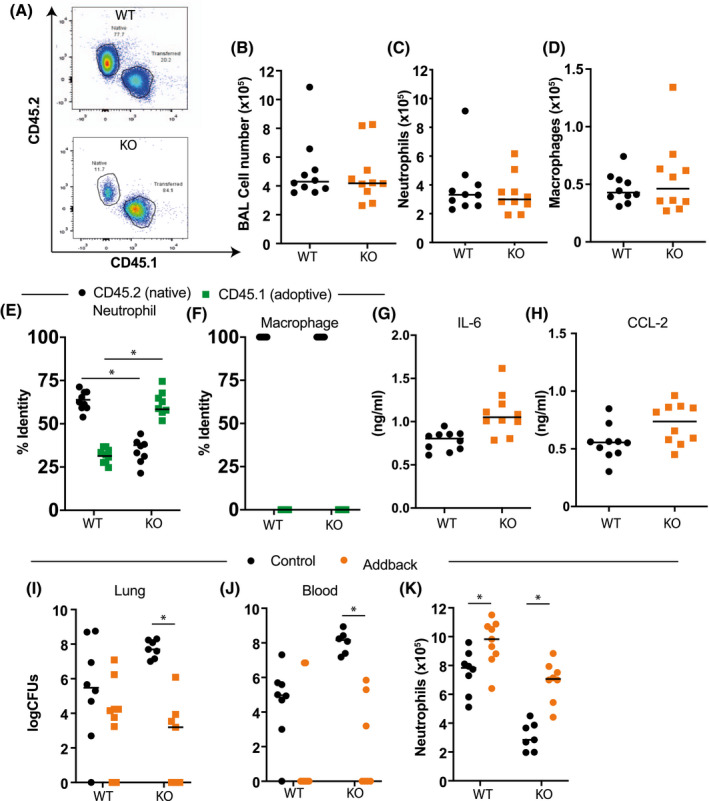
Functional EZH2 in Neutrophils is required for normal immune responses in the lung. A‐D, Bone marrow from control animals expressing CD45.1 (PEP3 mice) was used to purify neutrophils which were infused in the Ezh2^fl/fl^ (black circle) or LysM‐Ezh2^fl/fl^ (red square) animals by tail vein injection. Animals were exposed to nebulized LPS as above, and lung immune cell infiltration in bronchoalveolar lavage (BAL) determined by flow cytometry, macrophages (Ly6G‐, CD64+), and neutrophils (Ly6G+, CD11b+). (n = 10, median plotted, Mann‐Whitney *U*‐test not significant *P* > .05). E, The origin of lung neutrophils was measured by flow cytometry analysis of CD45.1 (donor) and CD45.2 (host). (n = 8, median plotted, Mann‐Whitney *U*‐test NS *P* > .05). F, Lung macrophage origin was also assessed by flow cytometry for CD45.1 status. The adoptive transfer rescued neutrophil recruitment to the lung and there were no adopted macrophages. G and H, Cytokine and chemokine concentration in BAL was measured by ELISA. (Mann‐Whitney U‐test; NS *P* > .05). I‐K, Mice (LysM‐Ezh2^fl/fl^ = KO) treated with adoptive transfer of neutrophils from CD45.1 wild‐type animals were compared to wild‐type control mice also infused with the same number of neutrophils. Animals were then inoculated with pneumococcus, and culled 48 hours later. The lung bacterial content (I) and peripheral blood bacterial content (J) were determined, and (K) lung neutrophil (Ly6G+, CD11b+) infiltration was assessed by flow cytometry analysis of BAL. (n = 6‐9, Kruskal‐Wallis with Dunn's multicomparison test (I&J), 2‐way ANOVA, post hoc Tukey's multicomparison test (K) *P < .05)

We next set out to define the role of EZH2 in neutrophil protection from pneumonia by using an adoptive transfer model, in which we restored EZH2 intact neutrophils to *LysM‐Ezh2^fl/fl^* targeted animals. Transferred neutrophils could be tracked by expression CD45.1 (Figure 5A). In initial proof‐of‐principle studies, following adoptive transfer, animals were exposed to aerosolized LPS.

In marked contrast to our earlier studies, there were no genotype‐specific differences in the number of BAL cells after LPS challenge (Figure 5B) or a change in the number of neutrophils (Figure 5C) or AMs (Figure 5D), indicating successful repopulation of recipient mice with fully functional neutrophils.

There was a marked change in the dominance of CD45 isoforms of the neutrophils between the *LysM‐Ezh2^fl/fl^* and *Ezh2^fl/fl^* control animals (Figure 5E). However, all the macrophages were CD45.2, and therefore of host animal origin (Figure 5F). We also noted no genotype‐specific differences in concentrations of IL‐6 (Figure 5G) or CCL2 (Figure 5H) in BAL. In order to show that neutrophil Ezh2 was essential to combat bacterial infection of the lung, we used this adoptive transfer model to test responses to intranasal infection with *S pneumoniae*. Here, we noted a significant reduction in lung bacterial burden of *LysM‐Ezh2^fl/fl^* mice after neutrophil add‐back, and no significant difference in the *Ezh2^fl/fl^* controls (Figure 5I). We also saw a reduction in bacterial recovery in blood in the addback animals, compared to the *LysM‐Ezh2^fl/fl^* host (Figure 5J). Further, BAL cell recovery after infection showed a significant increase following neutrophil addback in both genotypes, with the *LysM‐Ezh2^fl/fl^* mice showing an increase to control levels of BAL neutrophils (Figure 5K). Thus, we demonstrate complex and opposing effects of EZH2 deficiency in monocytic lineage cells, in which we observe an enhanced proinflammatory responses of macrophages, while loss of EZH2 in neutrophils impairs migration and responses to bacterial infection. Collectively, these responses hone the inflammatory response to facilitate a contained and sufficient host response to maximize bacterial clearance.

## 4 DISCUSSION

Regulation of innate immunity at mucosal surfaces is exquisitely sensitive to context, and requires interplay between resident immune surveillance cells, notably macrophages, epithelial cells, and recruited neutrophils.^16^ There has been interest in targeting epigenetic mechanisms to regulate the function of some hematopoietic cell malignancies, especially using drugs against the polycomb II complex.^17^ In addition, reports suggest targeting the enzymatic core of polycomb II, EZH2, impacts immune responses in the nonmalignant state.^15^ Therefore, it is timely to investigate the role of EZH2 in inflammation, and immunity.

We investigated the role of EZH2 in pulmonary mucosal inflammation, and for this used inflammatory challenge models targeting the lung, including nebulized lipopolysaccharide, (LPS), as well as two pulmonary infectious disease models (influenza A and pneumococcus). Initially, we targeted EZH2 in myeloid‐lineage cells using a *LysM‐cre* driver crossed to a floxed *Ezh2* line of mice, and this showed no significant change in cell surface markers after *Ezh2* deletion. Genetic deletion of EZH2 elicited similar proinflammatory cytokine responses, both in cultured macrophage cells and in vivo in the lung. Examining macrophages isolated from different compartments, we identified a gain in inflammatory response to canonical TLR activation. We were able to show that the effect of the EZH2 inhibitor UNC1999, a SAM‐binding site^17^ pharmacological inhibitor, was entirely dependent on the presence of EZH2, and thus confirmed the exquisite specificity of the tool compound. Our findings reveal a greater complexity in the macrophage responses dependent on EZH2, with increased late responses, and impaired early responses compared to previous reports.^15^ To investigate the basis for this change, we used a transcription factor activation assay and identified a specific impact of EZH2 on the TLR4 cascade, with no impact on the viral TLR3 response such that EZH2 has specific and selective action by inhibiting TLR4‐mediated proinflammatory pathways. This led us to hypothesize that the EZH2 effect may be specific to particular immune stimuli, with such specificity also likely as an explanation for differences reported here compared to those in earlier studies.^15^

*LysM* targets both neutrophil and macrophage populations. In order to distinguish macrophage effects from those of the neutrophils, we developed new lines of mice with *CX3CR1‐cre* targeting *Ezh2* deletion. This approach allows in vivo testing of innate responses, with neutrophil responses as part of the output. Although the CX3CR1 driver may also target NK cells and dendritic cells in the contact of acute bacterial infection, the primary drivers of response are macrophages and neutrophils. Using nebulized LPS, we saw a small increase in neutrophil infiltration resulting from EZH2 loss, but overall, the impact was surprisingly modest. However, LPS is a mimic of gram‐negative bacterial infection, which is less prevalent in the lung. So, we switched to infection with the gram‐positive bacterium *S pneumoniae*, a prevalent pulmonary pathogen and the commonest cause of community acquired pneumonia. These studies identified a major gain in immune response, with animals bearing macrophage‐targeted *Ezh2* having complete protection from bacteremia (ie, an extension of the pneumococcal infection to the bloodstream). Antibacterial gain was associated with increased numbers of both alveolar and interstitial macrophages. The gain in function here is far greater than anticipated from the LPS responses, and the gain in cell numbers suggests a more profound change in macrophage behavior resulting from EZH2 loss, possibly extending to reduced cell death in response to infection. Infection of control animals results in loss of macrophage numbers, a well‐described response, but this is reversed in the absence of EZH2.

As EZH2 played such a major role in the macrophage response to bacterial infection, we extended these studies to include the myeloid lineage more generally, as in the neutrophil population EZH2 is reported to exert a nongenomic role in cytoskeletal function, and motility.^19^ To achieve this, we used the *LysM‐cre* driver. Now, in the pneumococcal infection model, we observed impaired protection against infection, despite increased concentrations of inflammatory cytokines, and monocyte chemokines (CCL2). There were significantly reduced numbers of macrophages and neutrophils in the lung. The neutrophil migratory deficit was found to persist in vitro, with marked failure of the EZH2 deleted neutrophils to migrate toward a chemokine source, confirming a primary deficit in these manipulated cells.^19^ The reduction in macrophage numbers likely results from apoptosis resulting from excess bacteria, a well‐characterized response to bacterial sepsis in the lung.^23^

In order to investigate the contribution of the neutrophil deficit to the aberrant pneumococcal response in *LysM‐Ezh2^fl/fl^* targeted mice, we used a neutrophil‐adoptive transfer approach, using tail vein injection of bone marrow‐sourced neutrophils from CD45.1+ to both control, and *LysM‐Ezh2^fl/fl^* animals. The origin of the immune cells was tracked by using variant CD45 expression, and flow cytometry. There was a highly selective acquisition of transferred neutrophils, with no monocytes or macrophages in both recipient genotypes. Transfused CD45.1+ neutrophils were sufficient to restore the antibacterial response in the *LysM‐cre* mice, thus confirming that the defect in neutrophil recruitment to the lung was the likely primary cause of the impaired antibacterial response seen in the LysM‐cre‐directed EZH2 deletion. A possible explanation for this may be protein methyltransferase‐mediated activity of EZH2 on talin‐1 cleavage, which disrupts binding to F‐actin, leading to disrupted adhesion complex formation and aberrant cell migration.^19^ This is also consistent with more recent studies showing that disruption of talin1 methylation sites impairs transmigration of neutrophils across the peritoneal vascular epithelium during inflammation, an effect also mimicked by treatment with an EZH2 inhibitor.^24^

Bronchial epithelial cells are key effector cells in immunological responses to inflammatory challenge and pathogenic organisms, and our earlier work has identified a critical role in conferring time‐of‐day circadian responses to inflammatory challenge.^16^ However, we did not discover any difference in neutrophilic inflammatory responses in the epithelial EZH2‐null animals to either LPS or influenza infection. This suggests an unexpected specificity of cell type in the action of EZH2 on immunological responses.

In summary, we have characterized the role of EZH2 in myeloid cells during inflammatory challenges. While EZH2 in macrophages plays a specific role in controlling a TLR‐4:NF‐kB circuit, leading to increased cytokine production and augmented responses to inflammatory challenges and protection in in vivo pneumococcal exposure, loss of EZH2 function in neutrophils results in marked impairment of anti‐bacterial defense in the lung. Thus, loss of EZH2 in myeloid‐lineage cells has paradoxical and complex effects, leading to an apparent in vivo gain of function in macrophage cells, but defective migration and antibacterial responses of neutrophils. The implications of these studies need to be considered in the context of clinical use of EZH2 inhibitors. These are currently under evaluation for the treatment of various cancers, but our study suggests caution due to the immune consequences of targeting EZH2. It is possible that the inhibition of neutrophil migration resulting from EZH2 inhibition may have a therapeutic role in diseases associated with excess neutrophilic inflammation, such as adult respiratory distress syndrome.

## CONFLICT OF INTEREST

All authors have stated explicitly that there are no conflicts of interest in connection with this article.

## AUTHOR CONTRIBUTIONS

GBK, TH, DHD, TH, ASL, and DWR de‐signed research; GBK, TH, TGR, and NB performed research; JEG, ASL, and DWR contributed new reagents/analytic tools; GBK, TH, RGR, PD, ASL, and DWR analyzed data; and GBK, JEG, ASL, and DWR wrote the manuscript.

## Supporting information

Fig S1

Table S1

Supplementary Material
