# Supplementary material for: The histone methyltransferase Ezh2 restrains macrophage inflammatory responses
Source: FASEB J. 2021 Aug 31;35(10):e21843. doi: 10.1096/fj.202100044RRR (PMC8573545; doi:10.1096/fj.202100044RRR)

Figure S1: WT = Ezh2 fl/fl KO = LysM x Ezh2 fl/fl

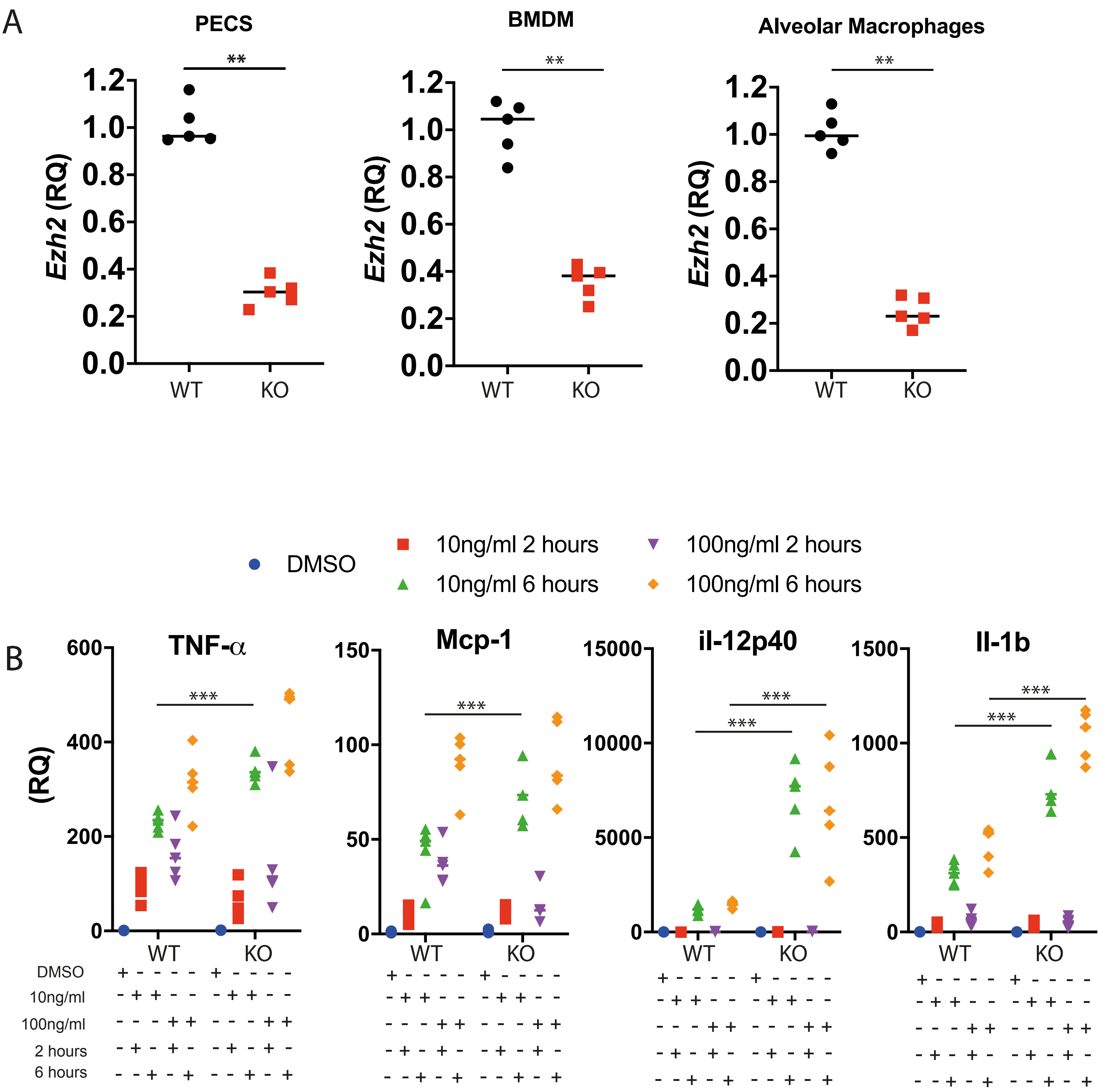

Figure S2

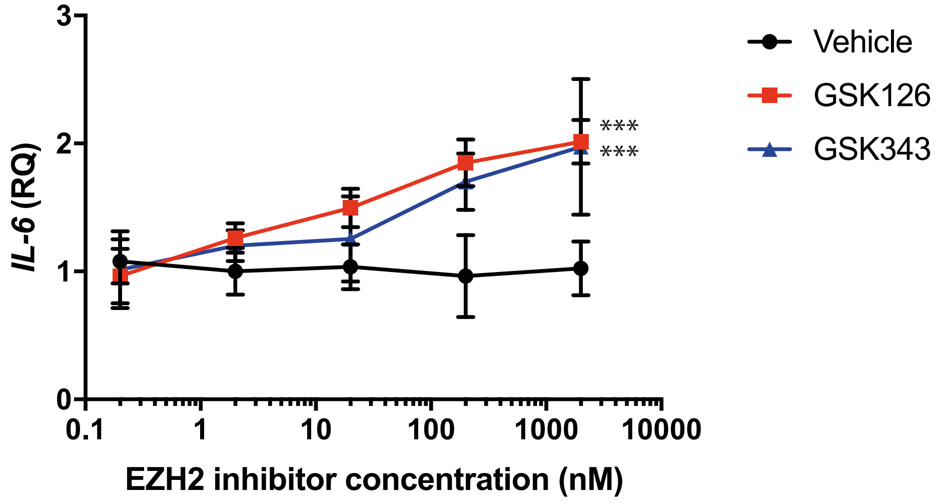
3
2
1
0
IL-6 (RQ)
0.1
1
10
100
1000
10000
EZH2 inhibitor concentration (nM)
***
***
Vehicle
GSK126
GSK343

Figure S3

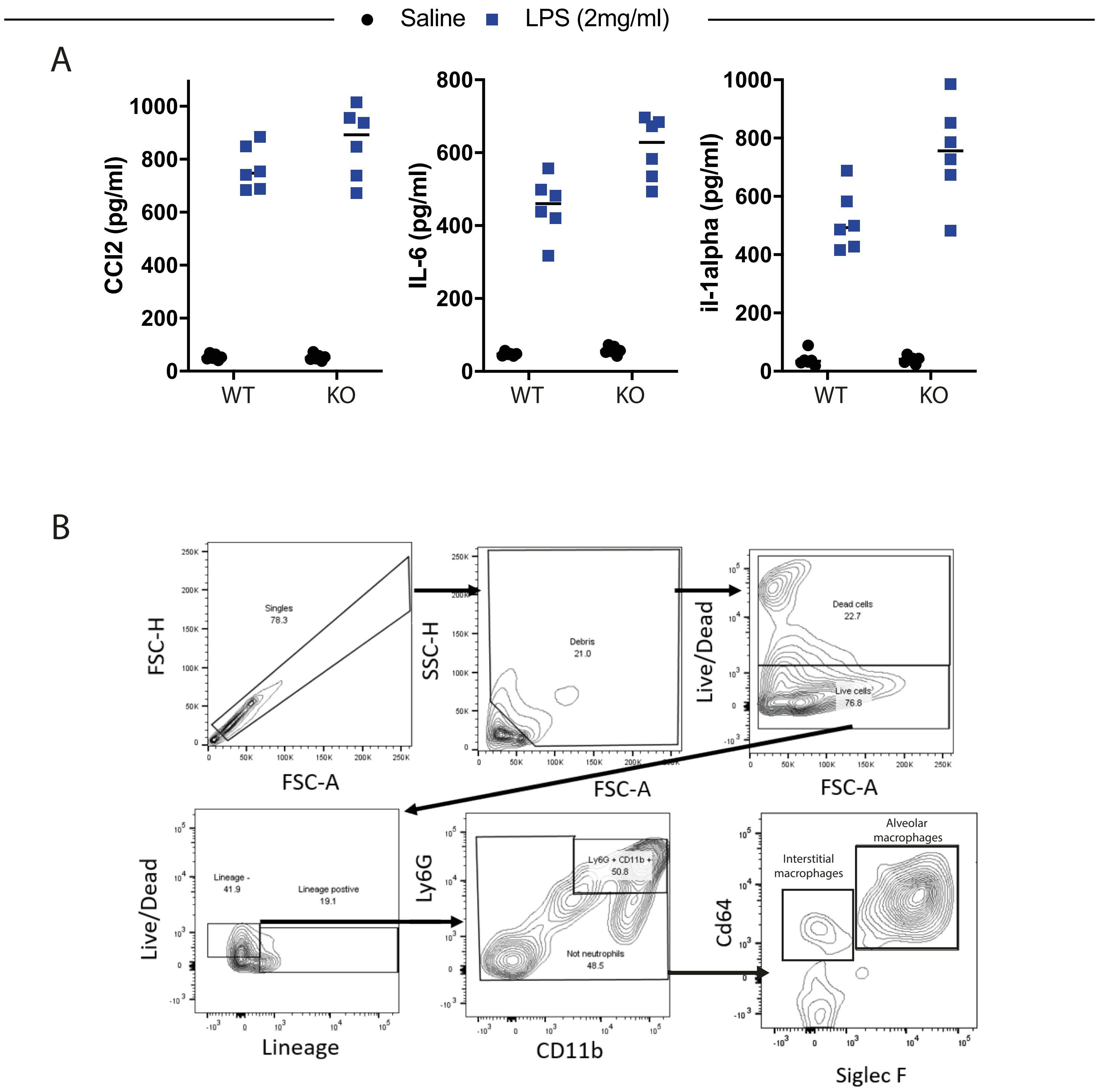

Saline
LPS (2mg/ml)
A
Ccl2 (pg/ml)
1000
800
600
400
200
0
WT
KO
IL-6 (pg/ml)
800
600
400
200
0
WT
KO
il-1alpha (pg/ml)
1000
800
600
400
200
0
WT
KO
B
FSC-H
Singles
78.3
FSC-A
SSC-H
Debris
21.0
FSC-A
Live/Dead
Dead cells
22.7
Live cells
76.8
FSC-A
Live/Dead
Lineage -
41.9
Lineage postive
19.1
Lineage
Ly6G
Ly6G + CD11b +
50.8
Not neutrophils
48.5
CD11b
Cd64
Interstitial macrophages
Alveolar macrophages
Siglec F

Figure S4. Influenza. WT = Ezh2 fl/fl KO = CCSP-icre x Ezh2 fl/fl

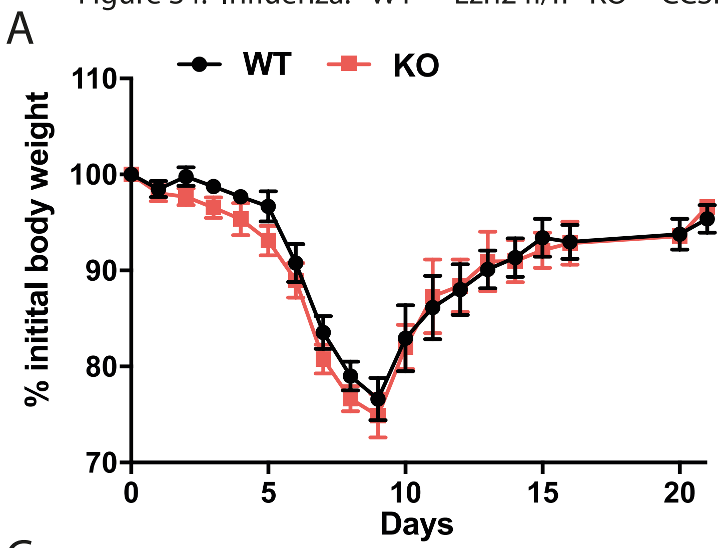

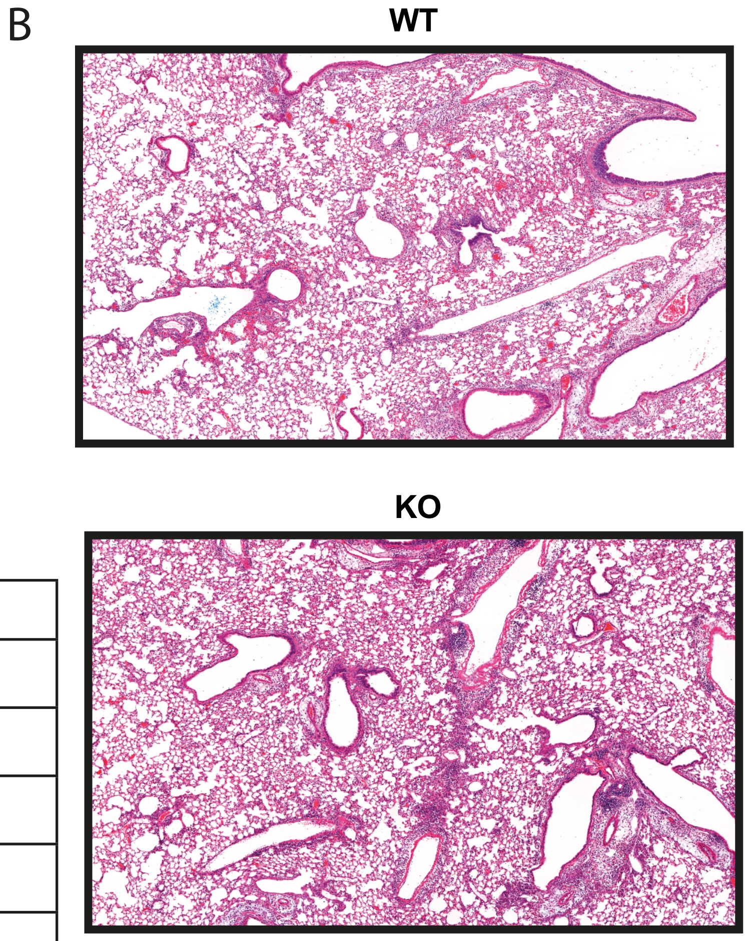

C

Histology Scoring

Score	WT	KO
1 = None	0	0
2 = Mild	2	2
3 = Moderte	2	1
4 = Severe	1	0
5 = Very severe	0	2
Total number of animals	5	5
Average score +/- SD	2.8 +/- 0.83	3.4 +/- 1.5

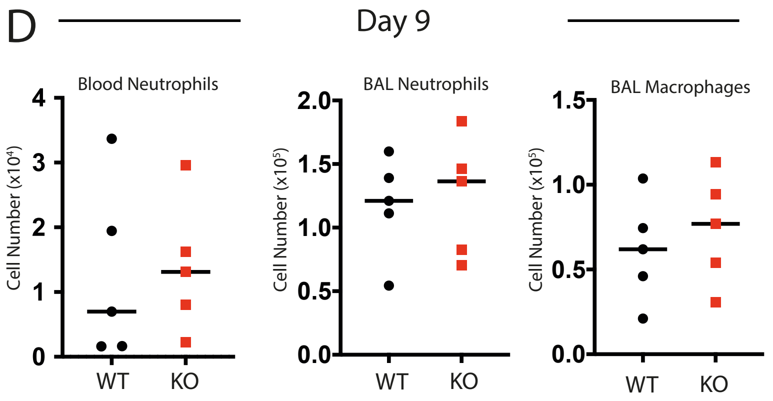

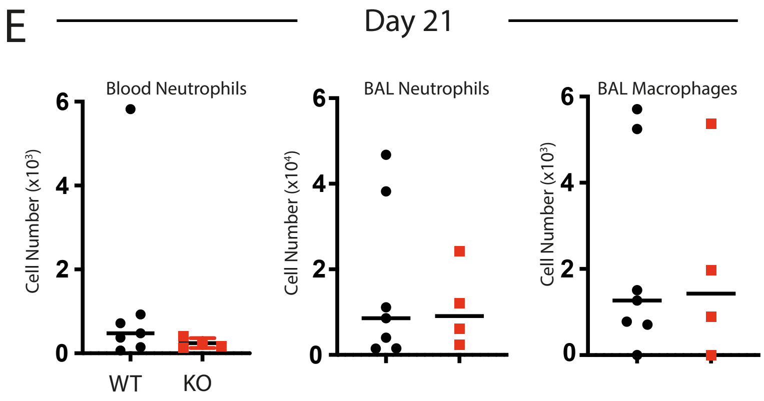

Supplement: Supplementary file 1 — Fig S1 [file FSB2-35-e21843-s001.pdf]
