# Supplementary material for: The histone methyltransferase Ezh2 restrains macrophage inflammatory responses
Source: FASEB J. 2021 Aug 31;35(10):e21843. doi: 10.1096/fj.202100044RRR (PMC8573545; doi:10.1096/fj.202100044RRR)
Supplement: Supplementary file 3 — Supplementary Material [file FSB2-35-e21843-s003.docx]

Supplementary legends

FigS1A

Transcript concentration for Ezh2, from peritoneal exudate cells (PECs), Bone marrow derived macrophages (BMDM), and alveolar macrophages. (n=5, median plotted, Mann-Whitney *U*-test **p<0.01, ***p<0.001)

Fig S1B

Isolated macrophages from bone marrow and treated with different doses of LPS for 2 or 6 hours shows that these cells are highly sensitive to time and concentration of treatment. Ex-vivo BMDMs from LysM-Ezh2^fl/fl^ macrophages and littermate controls were treated with LPS for 2 or 6 hours at a dose of 10 or 100ng/ml and the cytokine response assessed by RNA quantification. (n= 5, median plotted, 2-way ANOVA, post-hoc Tukey’s multi-comparison test. *** p<0.001).

Fig S2

EZH2 inhibitor dose response curve. Transcript abundance of Il-6 in ex-vivo bone marrow derived macrophages treated for 2hr with LPS (100ng/ml) following 48 hours incubation with one of the EZH2 inhibitors BSK343, GSK126, or DMSO control. IL-6 mRNA was quantified by qRT-PCR relative to Gaphd, and normalised to DMSO vehicle treated bone marrow derived macrophages. (n= 3, 2-way ANOVA, post-hoc Tukey’s multi-comparison test. *** p<0.001).

Fig S3

1. Pulmonary in-vivo inflammatory responses in CX3CR1-Ezh2^fl/fl^ (KO) and Ezh2^fl/fl^ (WT) mice. Mice were subject to nebulized LPS, and after 4 hours killed. The bronchoalveolar lavage (BAL) was analysed and the cytokine values are shown(n=6, median plotted, 2-way ANOVA, post-hoc Tukey’s multi-comparison test. p-value ns)
2. Flow cytometry gating strategy for quantification of pulmonary infiltrate

Fig S4 - Influenza responses in bronchial epithelial EZH2 null mice.

A) EZH2 was disrupted in bronchial epithelium using the CCSP-icre crossed with Ezh2fl/fl. CCSP-icre- Ezh2fl/fl (KO) and Ezh2fl/fl (WT) mice were infected with influenza, and responses tracked through time by measurement of body weight. The nadir at day 9 was followed by recovery, in both genotypes. Loss of EZH2 in these cells did not alter the trajectory of response to infection.

B) Histological analysis of lungs at Day 9 (H and E stain) did not reveal any differences by genotype.

C) At the peak of infection peripheral blood, and lung content of immune cell sets did not differ by genotype.

D, E) Analysis of blood and lung immune cell sets after recovery did not differ by genotype at day 9 or 21. Mann-Whitney *U*-test not significant P>0.05.
